# Standard Disinfecting Solutions Recommended by the State Board of Health of Pennsylvania

**Published:** 1887-04

**Authors:** 


STANDARD DISINFECTING SOLUTIONS RECOMMENDED BY THE STATE BOARD OF HEALTH OF PENNSYLVANIA.

1. STANDARD SOLUTION NO. 1.—Dissolve chloride of lime or bleaching powder of the best quality (containing at least twenty-five per cent. of available chlorine) in soft water in the proportion of four ounces to the gallon.

2. STANDARD SOLUTION NO. 2.—Dissolve corrosive sublimate and permanganate of potash in soft water in the proportion of two drachms of each salt to the gallon.

(NOTE.—1. This solution is highly poisonous. 2. It requires a contact of one hour to be efficient. 3. It destroys lead pipes. 4. It is without odor.)

3. STANDARD SOLUTION NO. 3.—To one part of Labarraque's Solution (*liquor sodæ chloratæ*, U. S. P.), of hypochlorite of soda and five parts of soft water.

(NOTE.—Competent authority has pronounced this superior to all other disinfectants.)

STANDARD SOLUTION NO. 4.—Dissolve corrosive sublimate in water in the proportion of four ounces to the gallon, and add one drachm of permanganate of potash to give color to the solution, as a precaution against poisoning, One fluid ounce of this solution to the gallon of water is sufficiently strong. Articles should be left in it for two hours.

(NOTE.—Corrosive sublimate solutions should be kept in wooden or crockery vessels.)

TO DISINFECT DISCHARGES FROM THE PATIENT.

Use standard solutions Nos. 1, 2 and 3, keeping a pint of the solution used constantly in the vessel, ready for any emergency. Let the discharges be passed directly into the solution, and then let a pint more of it be added, and allow the whole to stand for some time before being thrown into the sewer or being buried.

TO DISINFECT CLOTHING, TOWELS, NAPKINS, BEDDING AND SUCH TEXTILE FABRICS AS CAN BE WASHED.

Use standard solution No. 4, *one ounce to the gallon of water*, or use one gallon of solution No. 1 in nine gallons of water. Let the goods soak in the solution for at least two hours — better four hours — before they leave the room. Stir them up so that the solution gets all through them. After disinfection, boil the goods thoroughly.

FOR THE DISINFECTION OF WATER-CLOSETS, URINALS, SINKS AND CESS-POOLS.

CARBOLIC ACID SOLUTION.—Mix one pint of carbolic acid with two and a half gallons of water.

Standard solution No. 4, diluted with three parts of water, may also be used in the proportion of one gallon (of the solution) to every four (estimated) of the contents of the vault. Standard solution No. 1 would require to be used gallon for gallon of the material to be disinfected. Dry chloride of lime may be sprinkled over the contents of a privy, or standard solution No. 2 may be made up by the barrel, and four or five gallons be applied daily during an epidemic. In country places, an excellent plan, and probably the best plan to disinfect a privy, is to cover the contents with two or three feet of fresh earth. Every odor is effectually concealed by absorption in the earth.

TO DISINFECT THE SICK ROOM AFTER IT IS VACATED.

If it is possible let the room be thrown wide open for several days, for a thorough airing. If papered, let the paper be all removed with care Then let all the walls, the floor and the woodwork of the room, as well as the furniture, be washed with standard solution No. 4, one pint to four gallons of water, or of solution No. 1, a quarter of a pint to a gallon of water. Let this work be done most carefully, getting the solution into all the crevices. If any dust be present in the corners and crevices, wipe it out with a rag wet in the disinfecting fluid. *Don't stir it up with a brush or broom.* Last of all, whitewash the walls and the ceiling.

SULPHUR FUMIGATION

is believed in by many as very efficacious. Open wide all the drawers and closet doors. Hang on lines, opened up as much as possible, all the woolen articles which have been in the room during the sickness and which have not been disinfected and washed, then burn two pounds of sulphur for every thousand cubic feet in the room. Every opening into the room — flues, doors, windows, cracks and crevices — must be closed, except the door by which the disinfector is to escape. The sulphur is to be burned in an iron kettle or other vessel set in a tub containing a little water, to guard against fire. A little alcohol or kerosene must be poured upon the sulphur, by means of which it may be ignited. Leave the room quickly, for the fumes are highly poisonous when breathed, and close the door tightly. Let the room remain closed twenty-four hours or more. Then air thoroughly for several days.

